# Supplementary material for: Drug Repositioning Applied to Cardiovascular Disease in Mucopolysaccharidosis
Source: Life (Basel). 2022 Dec 12;12(12):2085. doi: 10.3390/life12122085 (PMC9784427; doi:10.3390/life12122085)
Supplement: Supplementary file 1 [file life-12-02085-s001.zip › life-1981374-Supplementary Figure S1.pdf]

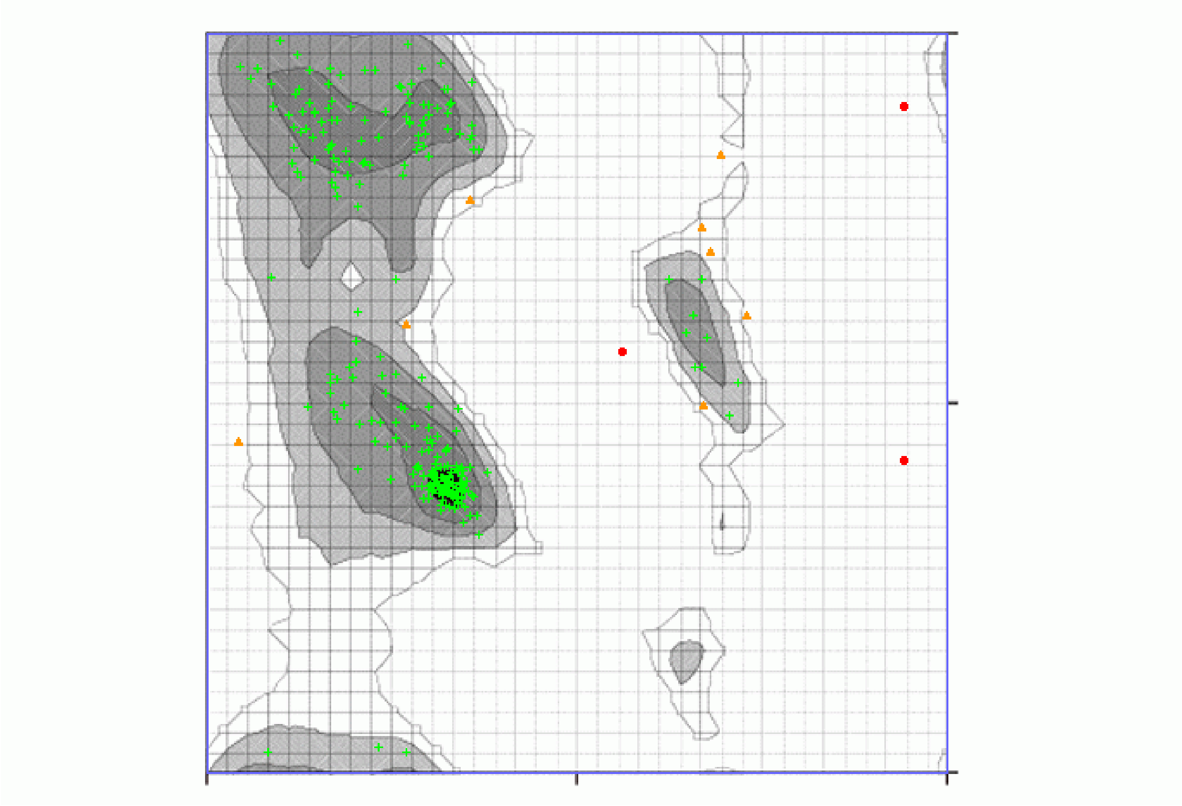

Supplementary Figure S1. Ramachandran plot of the homology model structure's quality. Black, dark grey, grey and light grey represent highly preferred conformations (Delta ≥ −2). White with black grid represent preferred conformations (−2 > Del-ta ≥ −4). White with grey grid represents questionable conformations (Delta < −4). Highly preferred observations shown as green crosses (255; 95.865%). Preferred observations shown as brown triangles (8; 3.008%). Questionable observations shown as red circles (3; 1.128%)
